# Electrical mapping of thermoelectric power factor in WO_3_ thin film

**DOI:** 10.1038/s41598-022-10908-3

**Published:** 2022-05-03

**Authors:** Sunao Shimizu, Tomoya Kishi, Goki Ogane, Kazuyasu Tokiwa, Shimpei Ono

**Affiliations:** 1 Materials Science Division, Central Research Institute of Electric Power Industry (CRIEPI), Kanagawa, 240-0196 Japan; 2 Faculty of Advanced Engineering, Tokyo University of Science, Tokyo, 125-8585 Japan

**Keywords:** Electronic devices, Thermoelectrics

## Abstract

With growing environmental awareness and considerable research investment in energy saving, the concept of energy harvesting has become a central topic in the field of materials science. The thermoelectric energy conversion, which is a classic physical phenomenon, has emerged as an indispensable thermal management technology. In addition to conventional experimental investigations of thermoelectric materials, seeking promising materials or structures using computer-based approaches such as machine learning has been considered to accelerate research in recent years. However, the tremendous experimental efforts required to evaluate materials may hinder us from reaping the benefits of the fast-developing computer technology. In this study, an electrical mapping of the thermoelectric power factor is performed in a wide temperature-carrier density regime. An ionic gating technique is applied to an oxide semiconductor WO_3_, systematically controlling the carrier density to induce a transition from an insulating to a metallic state. Upon electrically scanning the thermoelectric properties, it is demonstrated that the thermoelectric performance of WO_3_ is optimized at a highly degenerate metallic state. This approach is convenient and applicable to a variety of materials, thus prompting the development of novel functional materials with desirable thermoelectric properties.

## Introduction

Investigations on energy harvesting have become a mainstream in materials science, integrating different research disciplines such as physics, chemistry, electronics, and engineering^1–4^. This trend is motivated partly by the global requirement to develop advanced energy saving technologies^5,6^, aiming to reduce the current considerable carbon emission. Simultaneously, there is an urgent need to provide micro IoT (Internet of Things) sensors with stand-alone power systems^7,8^, the number of which will be skyrocketing in the coming decades. Thermoelectric energy conversion, which is a transformation of waste heat into electricity, has manifested itself as a promising CO_2_-free technology for generating electricity from environment^9^. In order to realize more useful and efficient thermoelectric energy conversion, new strategies have been proposed, such as flexible devices^10,11^, nano structures^12,13^, and ionic thermoelectric materials^14–16^. Furthermore, besides the continuous experimental efforts, computer-based approaches have emerged and been transforming traditional processes of experimental researches.

For the past decade, high-throughput calculations and machine learning have been advancing dramatically, demonstrating the indisputable utility of these approaches for the exploration of thermoelectric materials^17,18^. Computer-based materials research deductively or statistically investigate materials, structures, and compositions, to predict promising thermoelectric materials that should be evaluated experimentally. Thus, both experimental and computer-based approaches for exploration of thermoelectric materials should proceed together in tandem and feedback the results between them. However, considering the fast growth of computer-based science, experimental side would be required to accelerate; in general, evaluation of the thermoelectric properties requires great experimental effort because the figure of merit or power factor depends on mutually related parameters of the Seebeck coefficient *S* and the electrical conductivity *σ*, which drastically change with the carrier density *n*.

Recently, electrolyte gating techniques have been applied to a variety of materials and were found to be very effective for systematically controlling *n*^19^. By just applying several volts of the gate voltage *V*_G_, highly insulating electronic states are converted into conducting metallic states and even superconducting phases in oxides^20–22^, two dimensional materials^23^, and inorganic semiconductors^24^. Furthermore, continuous carrier doping using liquid electrolytes was found applicable to materials in which conventional methods such as bulk chemical doping were ineffective^25–27^. Thus, gate scanning of the thermoelectric properties in a wide temperature *T* and *V*_G_ (i.e., *n*) space would definitely help accelerate thermoelectric researches from an experimental side.

**Figure 1:**
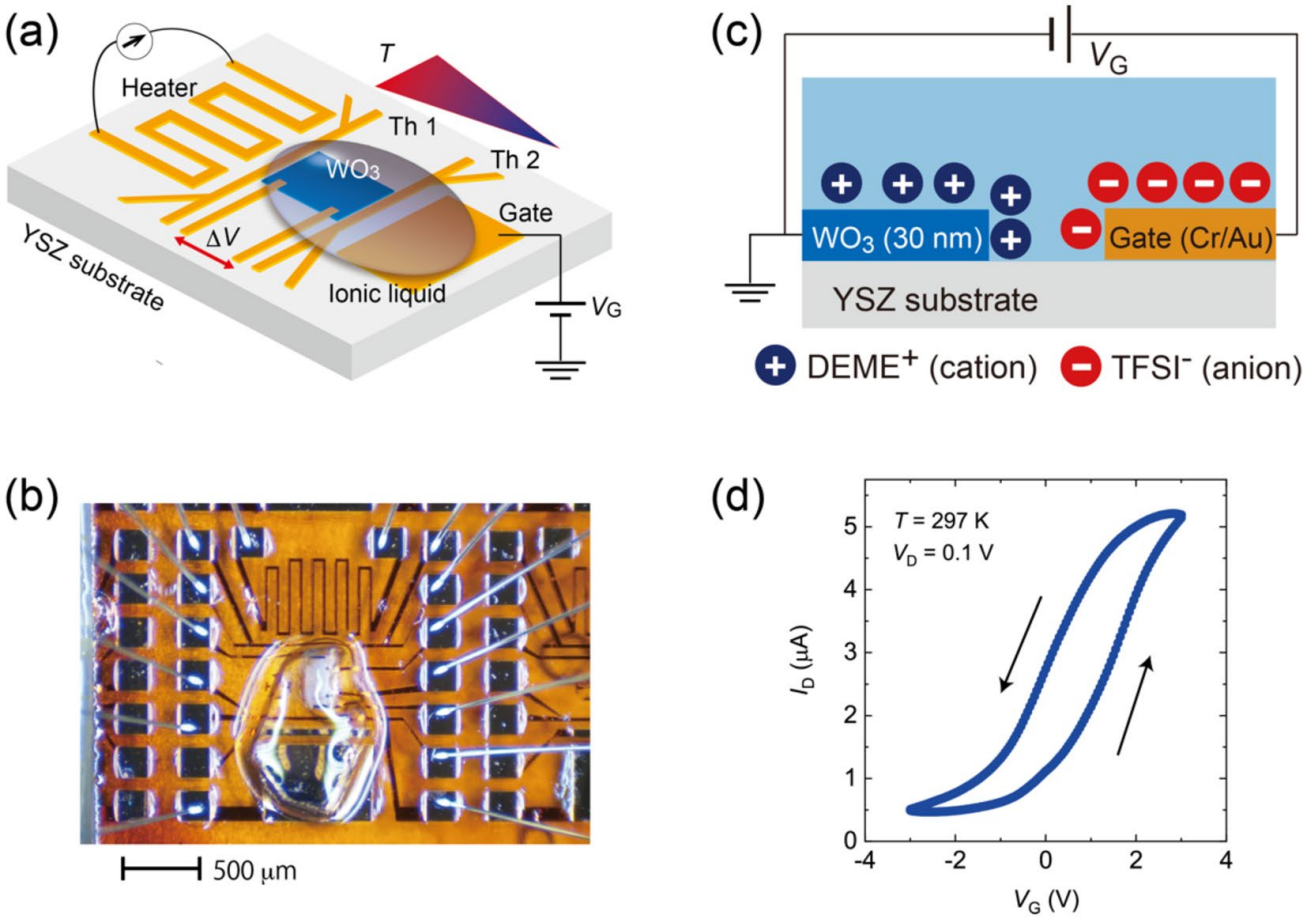
Structure and typical transistor operation of ion gated device based on WO_3_ thin film. (**a**) Schematic device structure to measure the Seebeck effect under gate voltage *V*_G_. In this configuration, the resistance and the Seebeck coefficient are simultaneously measured. Here, Th1(Th2), *T*, and Δ*V* stand for the resistive thermometer, temperature, and thermoelectric voltage, respectively. A drop of an ionic liquid was deposited to cover the WO_3_ channel and the gate electrode. See the “Experimental section” for more details of the device structure. (**b**) Optical image of WO_3_ device with ionic liquid on top. (**c**) Schematic diagram of alignment of ions under *V*_G_. When a positive *V*_G_ is applied to the gate electrode, the cations align on the surface of the WO_3_ thin film to form the electric double layer, which could trigger both the electrostatic carrier accumulation and electrochemical doping. (**d**) Typical transfer characteristics (drain- source current *I*_D_ versus *V*_G_). The values of *I*_D_ increases with increasing *V*_G_, showing that the electron carriers are doped under positive *V*_G_. The drain voltage *V*_D_ was 0.1 V.

In this study, we present the gate control of thermoelectric properties in WO_3_, a functional oxide semiconductor that has been attracting attention^28–34^. Although thermoelectric measurements in WO_3_ and related materials have been conducted especially in the last decade^28,35^, basic characteristics such as *n* and *T* dependences have not been investigated systematically, which makes WO_3_ the best choice to study the effect of electrolyte gating. We synthesized WO_3_ thin films with the thickness of 30 nm on a substrate of yttria stabilized zirconia (YSZ). The synthesis process and the X-ray diffraction (XRD) data (see Supplementary Figure S1) were reported elsewhere^36^. An electric double layer transistor structure was fabricated by depositing a drop of an ionic liquid on the WO_3_ thin film, as shown in Fig. 1a and b. We systematically investigated the thermoelectric power factor of WO_3_ and mapped the data for a wide range of *T* and *V*_G_. The results demonstrate how the thermoelectric properties develop against carrier doping, providing a solid guideline for the investigation of much higher thermoelectric performance in WO_3_.

## Results and discussion

The details of the device structure and the concept behind its fabrication are summarized in Fig. 1. The device was patterned using the standard photolithography technique. As seen in Fig. 1a, b, and Supplementary Figure S2, a drop of the ionic liquid covered the WO_3_ thin film and the gate electrode, forming the electric double layer on the surface of WO_3_. Figure 1c schematically shows that the application of the positive gate bias causes the cations align on the surface of WO_3_, inducing a strong electric field on the interface. A typical transfer characteristic (*V*_G_ dependence of the drain source current *I*_D_) at 297 K is given in Fig. 1d, suggesting that electron carriers were induced under the positive gate bias. The hysteresis was observed possibly due to the slow re-formation of ions against *V*_G_. Two resistive thermometers and a heater were prepared by the metal evaporation on the same substrate. The heater was placed in the immediate vicinity of WO_3_ to induce a thermal gradient along the channel. The local temperatures were monitored using the two thermometers, Th1 and Th2, and the thermoelectric voltage Δ*V* was measured using the electrodes connected to both ends of the channel (See Supplementary Notes). Therefore, the device structure prepared for this study allows for the evaluation of thermoelectric properties under the application of gate electric field (See “Experimental section”)^37^.

**Figure 2:**
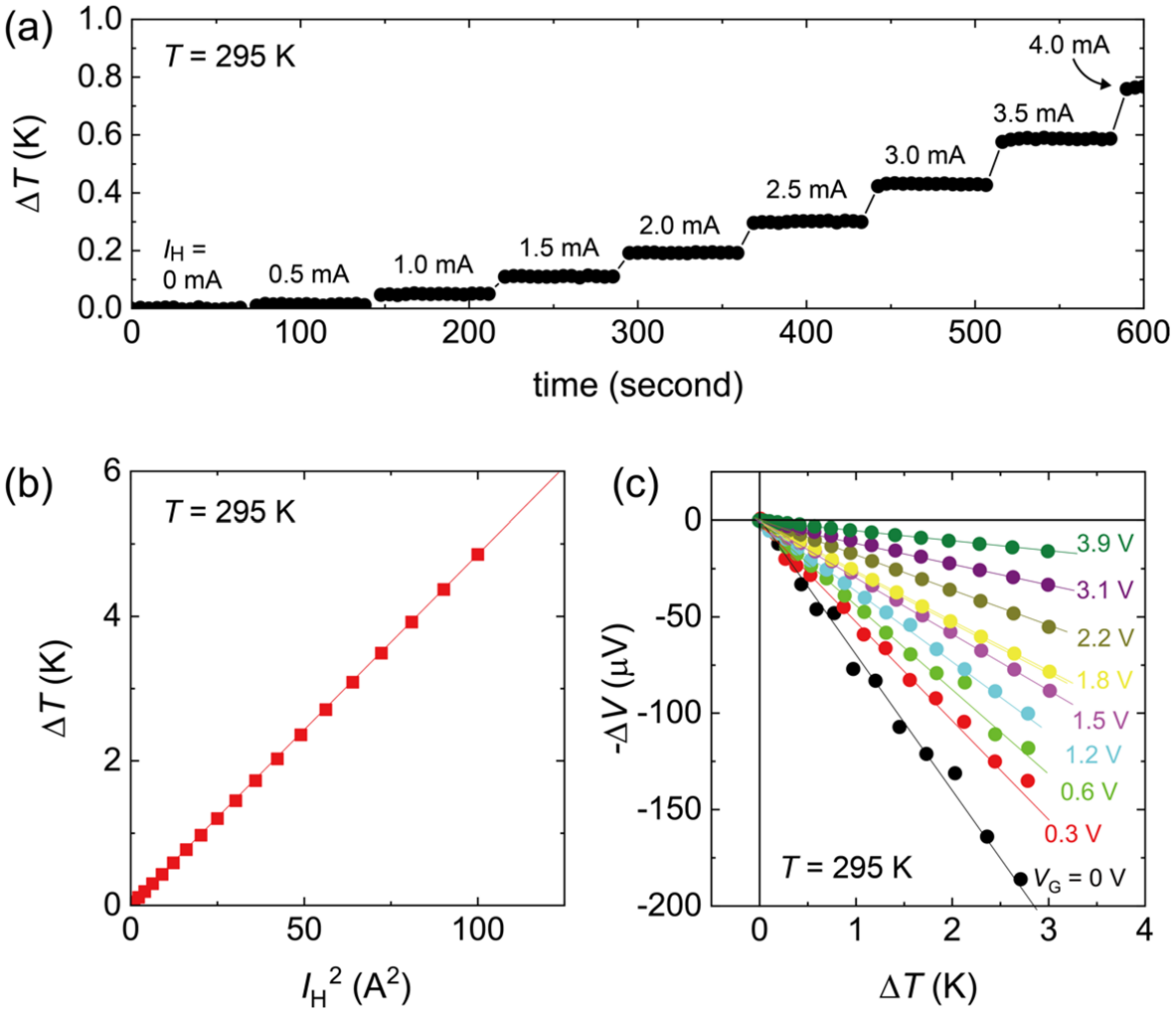
On-chip thermoelectric measurements of WO_3_ thin film. (**a**) Temperature difference Δ*T* for different heater current *I*_H_. When *I*_H_ was applied to the heater in Fig. 1a, Δ*T* was induced between the edges of the channel due to the Joule heating of the heater. The values of Δ*T* increased with increasing *I*_H_. (**b**) Heater power dependence of Δ*T*. The values of Δ*T* linearly increased against *I*_H_^2^, confirming that the temperature gradient on the sample is solely attributed to the Joule heating of the heater. (**c**) Thermoelectric voltage Δ*V* under Δ*T* in WO_3_ thin film. The measurements were performed at 295 K. The slope of the Δ*V* − Δ*T* plot was systematically suppressed with increasing *V*_G_.

The thermoelectric effect was measured in this on-chip device structure (Fig. 1a and b) and systematically controlled under the gate bias. Figure 2a shows the temperature difference Δ*T* between Th1 and Th2 (see Supplementary Figure S2) for different values of the heater current *I*_H_. The values of Δ*T* showed a stepwise increase with increasing *I*_H_; Δ*T* was stable for a fixed value of *I*_H_. It was also confirmed from Fig. 2b that Δ*T* was proportional to *I*_H_^2^, validating that the thermal gradient on the WO_3_ thin film was induced solely by the Joule heating of the heater. Figure 2c shows the Δ*V* − Δ*T* plot of the WO_3_ thin film for different *V*_G_ at 295 K. The values of Δ*V* linearly increased with Δ*T*, indicating that the thermoelectric effect was correctly measured. The slope of the Δ*V* − Δ*T* plot was continuously suppressed with increasing *V*_G_, which shows that the absolute values of *S* were suppressed. It is worth noting that the change of the thermoelectric response seen in Fig. 2c is consistent with the gate induced *I*_D_ modulation seen in Fig. 1d. Generally, in semiconductors, |*S*| is suppressed by the shift of the Fermi level to higher energy regions^38^. Thus, both results in Figs. 1d and 2c are the direct outcome of the gate-induced electron doping into the WO_3_ thin film.

**Figure 3:**
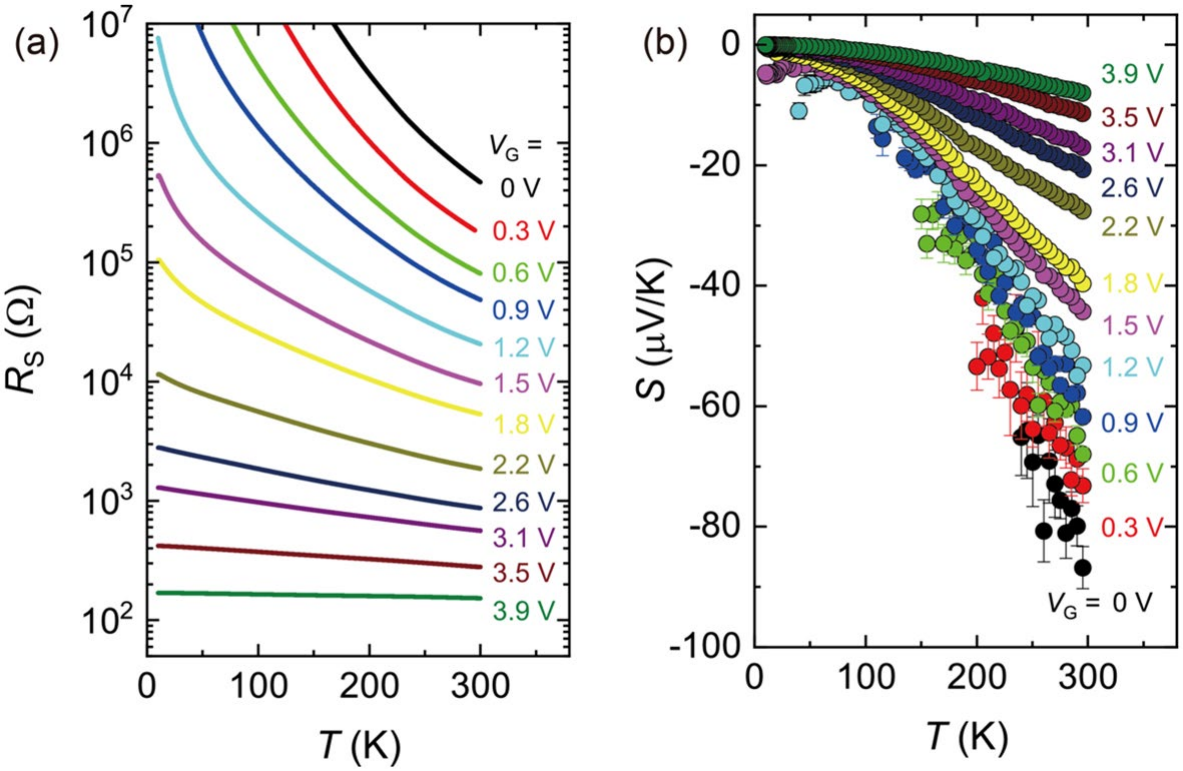
Gate induced transition from insulator to metal in WO_3_ thin film. (**a**) Temperature *T* dependence of sheet resistance *R*_s_ under gate bias. The values of *R*_s_ are suppressed by the application of gate voltage *V*_G_. *R*_s_ showed insulating behavior at *V*_G_ = 0 V, whereas a flat *T* dependence was observed at *V*_G_ = 3.9 V. (**b**) *T* dependence of Seebeck coefficient *S* under gate bias. The values of *S* were gradually suppressed with increasing *V*_G_ in the entire measured *T* region. For low *V*_G_ values, *S* was obtained only at high temperatures because the resistance between the voltage probe for Δ*V* including the contact resistance was too high (> ~ MΩ). For large *V*_G_ values, *S* approached zero with decreasing *T*, which is typical behavior in metallic semiconductors. The error bars correspond to the standard error in the linear fitting of Δ*V*–Δ*T* plot.

The gate-induced carrier doping is much clearly illustrated in the *T* dependence measurements of the electrical and thermoelectric transports. Figure 3a shows the *T* dependence of the sheet resistance *R*_s_ of the WO_3_ thin film for different *V*_G_. When *V*_G_ = 0 V, *R*_s_ showed a large value of ~ 0.5 MΩ at 295 K and semiconducting behavior against *T*. With increasing *V*_G_, *R*_s_ systematically decreased, accompanied by a transition from an insulating to a metallic state^33,36^. The lowest value of *R*_s_ obtained here was ~ 200 Ω for *V*_G_ = 3.9 V, which is comparable to the previous reports on ion-gated WO_3_^30,31,36^. As shown in Fig. 3b, we simultaneously evaluated the thermoelectric response of WO_3_ with the measurement of *R*_s_. The values of *S* were gradually suppressed with increasing *V*_G_ in the entire measured *T* range. For low *V*_G_ values, *S* was obtained only at high temperatures because the resistance between the voltage probe for Δ*V* measurements including the contact resistance became too high (> ~ MΩ), which made it difficult to reliably measure Δ*V* at low temperatures. For large *V*_G_ values, *S* approached zero with decreasing *T*, which is typical behavior in metallic semiconductors^38^. Here we note that the systematic modulation of *S* in bulk WO_3_ has never been realized by conventional chemical doping method; the electric double layer doping is a unique approach to explore the thermoelectric properties of semiconductors.

**Figure 4:**
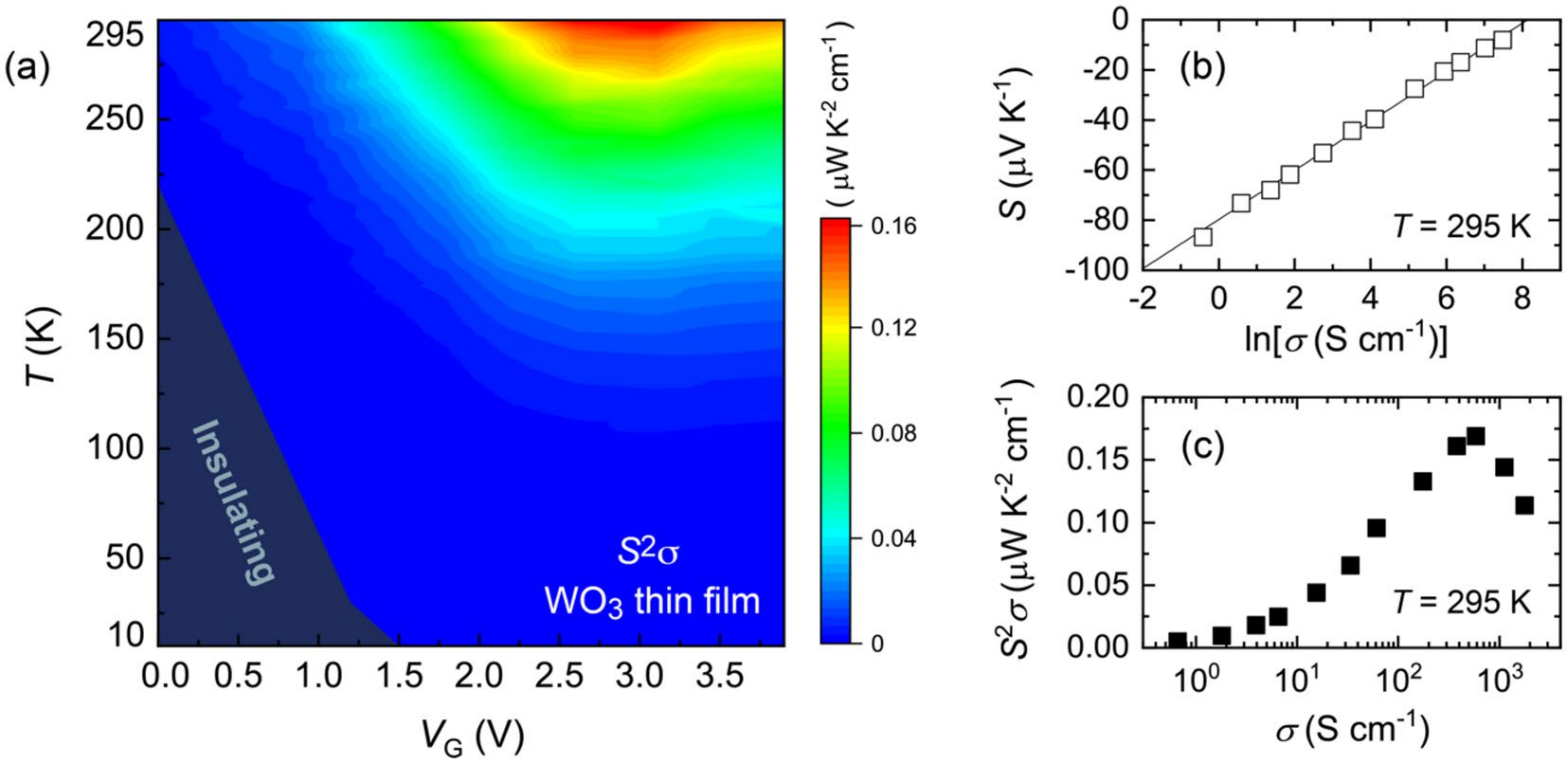
Optimization of thermoelectric power factor in WO_3_ thin film. (**a**) Mapping of thermoelectric power factor *S*^2^*σ* against temperature *T* and gate voltage *V*_G_. The value of *S*^2^*σ* increased with increasing *V*_G_ and showed a maximum at *V*_G_ ~ 3.1 V. (**b**) Evolution of *S* against electrical conductivity *σ*. The values of *S* change linearly with ln(*σ*), which holds for a variety of semiconductors^45^. (**c**) Optimization of *S*^2^*σ* of WO_3_ at room temperature.

The overall thermoelectric property of the WO_3_ thin film is electrically visualized in a wide *T* and *V*_G_ space. Figure 4a shows the contour plot of the thermoelectric power factor *S*^2^*σ*, using the data from Fig. 3. The electrical conductivity *σ * was estimated as *σ*  = 1/(*d* × *R*_s_), where *d* is the thickness of the thin film, ~ 30 nm. Here, we assume that the carrier accumulation layer thickness is the same with *d*. Generally, in field effect transistors, only the topmost layer as thin as several nanometers is affected by the gate electric field. However, it has been reported that electrolyte gating can uniformly dope electrons into the whole WO_3_ thin film, even for thick films having a thickness of 70 nm^30,33^. This suggests that the electron doping does not proceed electrostatically but occurs electrochemically in the ion-gated WO_3_ thin film^30,33^.

The systematic modulation of the thermoelectric property in Fig. 4a suggests the continuous change in the electronic structure for the WO_3_ thin film. In the low *V*_G_ region, *R*_s_ increased with decreasing *T*, as shown in Fig. 3a, suggesting that the WO_3_ thin film is nondegenerate with an activation energy *E*_a_^39,40^. The relationship between *σ* and *T* can be expressed with an Arrhenius type equation,

1 $$\sigma = \sigma_{0} {\text{exp}}\left( { - \frac{{E_{{\text{a}}} }}{{k_{{\text{B}}} T}}} \right)$$

where *σ*_0_ is a pre-exponential factor, and *k*_B_ is the Boltzmann constant. We applied Eq. (1) to the experimental curve of *R*_s_ for the most resistive state with *V*_G_ = 0, according to the analysis conducted by Mattoni *et al*^40^. The estimated value of *E*_a_ was ~ 106 meV (see Supplementary Figure S5), which is one order of magnitude smaller than the insulating gap of WO_3_, ~ 3.0 eV^28,41–43^. This suggests that, at *V*_G_ = 0 V, a shallow donor level exists below the conduction band bottom due to oxygen vacancies in the film^40^. On the other hand, in the large *V*_G_ region, *R*_s_ was drastically suppressed and showed a flat *T* dependence, as shown in Fig. 3a. A systematic trend can be also seen in the thermoelectric response in Fig. 3b. The absolute value of *S* was gradually suppressed with increasing *V*_G_ due to the increase in the carrier doping level. These suggest that the WO_3_ thin film was highly doped and degenerate.

Our electrochemical approach modifies *S* of WO_3_ in a similar manner with the bulk chemical doping on other inorganic semiconductors. The relationship between *S* and *σ* in doped semiconductors is described by the following expression^44–46^ as

2 $$S = - \frac{{k_{{\text{B}}} }}{e} \times \left( { - \ln \sigma + \ln \left[ {N_{{\text{c}}} e\mu } \right] + \frac{5}{2} + r} \right)$$

where *N*_c_, *μ*, and *r*, are the effective density of states for the conduction band, the carrier mobility, and the scattering parameter, respectively. Equation 2 suggests that *S* is proportional to ln* σ* when the *n* dependence of μ is moderate. This condition would hold in transition metal oxides with low carrier mobility at room temperature^47,48^. Actually, the linear relationship between *S* and ln* σ* has been confirmed for a variety of semiconductors although the slope of the plot often varies from *k*_B_/*e* for each material^45^. Figure 4b shows the change in *S* as a function of *σ* for the WO_3_ thin film, which demonstrated that a good linearity between *S* and ln* σ* reasonably held in WO_3_ as well. When we plot *S*^2^*σ* as a function *σ*, a peak was observed at *σ*  = 585 S cm^-1^, as shown in Fig. 4c. The value of *S*^2^*σ* was modulated and optimized through the continuous carrier modulation from an insulating to a metallic region, which has never been found in the studies of bulk WO_3_. Here, it would be noted from Fig. 4a that the highest *S*^2^*σ* would exist above room temperature. Expanding this approach toward much higher temperatures would be more useful for the investigation of novel thermoelectric materials.

## Conclusions

In conclusion, we revealed the systematic evolution of thermoelectric properties of WO_3_ against electrochemical carrier doping. Ion gating is a versatile technique in carrier doping and continuously modulated *n* of the WO_3_ thin film. The value of *S*^2^*σ* was optimized to have ~ 0.16 μW K^-2^ cm^-1^ in a highly electron-doped region, where WO_3_ exhibited the metallic electrical and thermoelectric transports. These findings could serve as an important guideline for exploring much higher thermoelectric performance in bulk WO_3_ and its related materials such as nanocrystalline WO_3_ and WO_3_-based composites^28,49,50^. The current approach, the *T* − *V*_G_ (i.e., *n*) mapping of *S*^2^*σ*, is applicable to various functional materials other than WO_3_ as well. Its synergetic combination with other approaches such as computation and machine learning would make that a powerful tool, leading to the emergence of high performance multifunctional thermoelectric materials hidden in unexplored materials groups.

## Experimental section

### Thin film growth

The hexagonal WO_3_ film were grown on a YSZ(111) substrate by evaporating tungsten via RF sputtering with a RF power of 30 W. We followed the same procedure that was reported elsewhere^36^. The O_2_ partial pressure was controlled at ~ 2.2 mTorr in the growth chamber, and the Ar partial pressure was maintained at ~ 8 mTorr. Supplementary Figure S1 in Supplementary Information shows the XRD curves of out-of-plane and in-plane configurations, which confirmed that the hexagonal structure of WO_3_ was realized. The thickness of the film was controlled by the deposition time and was estimated with X-ray reflectometry. The film thickness was estimated to be ~ 30 nm.

### Fabrication of ion gated device

To control the electron carrier density in the WO_3_ thin film, an electric double layer transistor structure was fabricated, which is schematically shown in Fig. 1a. The optical image of the device is shown in Fig. 1b and Supplementary Figure S2. The channel length and width were 300 μm and 100 μm, respectively. The electrodes and the channel were patterned using standard photolithography techniques. All the electrodes required for the ion gating experiments were prepared by the evaporation of Cr/Au with the thickness of 3/35 nm. In addition, the resistive thermometers, Th1 and Th2, and a heater were also prepared using the same procedure. A small amount of an ionic liquid *N*,*N*-dimethyl-*N*-(2-methoxyethyl)-*N*-methylammonium bis-(trifluoromethylsulfonyl)-imide (DEME-TFSI) was deposited to cover both the channel and the gate electrode in order to conduct gating experiments with a side gate configuration. DEME-TFSI has the glass transition temperature at 182 K, below which the polarization of ions is fixed and cannot be modulated by *V*_G_^51,52^. Throughout the experiments, *V*_G_ was applied and changed at 300 K.

### Thermoelectric measurements

As schematically shown in Fig. 1a, we prepared a heater in close vicinity of one end of the WO_3_ channel; it produced a thermal gradient −∇*T* from the heater to the other edge of the channel^37^. Two resistive thermometers, Th1 and Th2, monitored the *T* differences, which was induced by the Joule heating of the heater (See Fig. 2). The values of *S* were estimated as −*E*/|∇*T* |, where *E* is the electric field induced through the Seebeck effect, by measuring the thermoelectric voltage between both ends of the WO_3_ channel. The calibration of resistive thermometers is described in detail in Supplementary Notes.

## Supplementary Information

Supplementary Information.

## References

[1] Wang H, Park J. Do, Ren ZJ (2015). Practical energy harvesting for microbial fuel cells: A review. Environ. Sci. Technol..

[2] Russ B, Glaudell A, Urban JJ, Chabinyc ML, Segalman RA (2016). Organic thermoelectric materials for energy harvesting and temperature control. Nat. Rev. Mater..

[3] Lee JH (2016). All-in-one energy harvesting and storage devices. J. Mater. Chem. A.

[4] Vallem V, Sargolzaeiaval Y, Ozturk M, Lai Y-C, Dickey MD (2021). Energy harvesting and storage with soft and stretchable materials. Adv. Mater..

[5] Bizon N, Tabatabaei NM, Blaabjerg F, Kurt E (2017). Energy Harvesting and Energy Efficiency: Technology, Methods, and Applications.

[6] Bai Y, Jantunen H, Juuti J (2018). Energy harvesting research: The road from single source to multisource. Adv. Mater..

[7] Kumar S, Tiwari P, Zymbler M (2019). Internet of Things is a revolutionary approach for future technology enhancement: A review. J. Big Data.

[8] Hu G, Edwards H, Lee M (2019). Silicon integrated circuit thermoelectric generators with a high specific power generation capacity. Nat. Electron..

[9] Petsagkourakis I (2018). Thermoelectric materials and applications for energy harvesting power generation. Sci. Technol. Adv. Mater..

[10] Yang C (2017). Transparent flexible thermoelectric material based on non-toxic earth-abundant p-type copper iodide thin film. Nat. Commun..

[11] Rösch AG (2021). Fully printed origami thermoelectric generators for energy-harvesting. npj Flex. Electron..

[12] Heremans JP, Dresselhaus MS, Bell LE, Morelli DT (2013). When thermoelectrics reached the nanoscale. Nat. Nanotechnol..

[13] Blackburn JL, Ferguson AJ, Cho C, Grunlan JC (2018). Carbon-nanotube-based thermoelectric materials and devices. Adv. Mater..

[14] Li T (2019). Cellulose ionic conductors with high differential thermal voltage for low-grade heat harvesting. Nat. Mater..

[15] Pu S (2020). Thermogalvanic hydrogel for synchronous evaporative cooling and low-grade heat energy harvesting. Nano Lett..

[16] Han C-G (2020). Giant thermopower of ionic gelatin near room temperature. Science.

[17] Prashun G, Vladan S, Eric ST (2017). Computationally guided discovery of thermoelectric materials. Nat. Rev. Mater..

[18] Wang T, Zhang C, Snoussi H, Zhang G (2019). Machine learning approaches for thermoelectric materials research. Adv. Funct. Mater..

[19] Bisri SZ, Shimizu S, Nakano M, Iwasa Y (2017). Endeavor of iontronics: From fundamentals to applications of ion-controlled electronics. Adv. Mater..

[20] Shimotani H (2007). Insulator-to-metal transition in ZnO by electric double layer gating. Appl. Phys. Lett..

[21] Ueno K (2008). Electric-field-induced superconductivity in an insulator. Nat. Mater..

[22] Bollinger AT (2011). Superconductor-insulator transition in La_2-x_Sr_x_CuO_4_ at the pair quantum resistance. Nature.

[23] Ye JT (2012). Superconducting dome in a gate-tuned band insulator. Science.

[24] Yamamoto HM (2013). A strained organic field-effect transistor with a gate-tunable superconducting channel. Nat. Commun..

[25] Krüger M, Buitelaar MR, Nussbaumer T, Schönenberger C, Forró L (2001). Electrochemical carbon nanotube field-effect transistor. Appl. Phys. Lett..

[26] Yoshida M (2016). Gate-optimized thermoelectric power factor in ultrathin WSe_2_ single crystals. Nano Lett..

[27] Shimizu S (2016). Thermoelectric detection of multi-subband density of states in semiconducting and metallic single-walled carbon nanotubes. Small.

[28] Zheng H (2011). Nanostructured tungsten oxide - Properties, synthesis, and applications. Adv. Funct. Mater..

[29] Katase T, Onozato T, Hirono M, Mizuno T, Ohta H (2016). A transparent electrochromic metal-insulator switching device with three-terminal transistor geometry. Sci. Rep..

[30] Altendorf SG (2016). Facet-independent electric-field-induced volume metallization of tungsten trioxide films. Adv. Mater..

[31] Nishihaya S (2016). Evolution of insulator-metal phase transitions in epitaxial tungsten oxide films during electrolyte-gating. ACS Appl. Mater. Interfaces.

[32] ViolBarbosa C (2016). Transparent conducting oxide induced by liquid electrolyte gating. Proc. Natl. Acad. Sci. U. S. A..

[33] Leng X (2017). Insulator to metal transition in WO_3_ induced by electrolyte gating. npj Quantum Mater..

[34] Onozato T, Nezu Y, Cho HJ, Ohta H (2019). Fast operation of a WO_3_-based solid-state electrochromic transistor. AIP Adv..

[35] Cerretti G, Balke B, Kieslich G, Tremel W (2018). Towards higher zT in early transition metal oxides: Optimizing the charge carrier concentration of the WO_3-x_ compounds. Mater. Today Proc..

[36] Wu PM (2015). Synthesis and ionic liquid gating of hexagonal WO_3_ thin films. Appl. Phys. Lett..

[37] Xing H, Zhang P, Zeng H (2020). Thermoelectric probe of defect state induced by ionic liquid gating in vanadium dioxide. Appl. Phys. Lett..

[38] Goldsmid HJ (2010). Introduction to Thermoelectricity.

[39] Goulding MR, Thomas CB (1979). The transport properties of amorphous films of tungstic oxide, sublimed under different conditions. Thin Solid Films.

[40] Mattoni G, Filippetti A, Manca N, Zubko P, Caviglia AD (2018). Charge doping and large lattice expansion in oxygen-deficient heteroepitaxial WO_3_. Phys. Rev. Mater..

[41] Chen B (2013). The band structure of WO3 and non-rigid-band behaviour in Na_0.67_WO_3_ derived from soft x-ray spectroscopy and density functional theory. J. Phys. Condens. Matter..

[42] Lee T, Lee Y, Jang W, Soon A (2016). Understanding the advantage of hexagonal WO_3_ as an efficient photoanode for solar water splitting: A first-principles perspective. J. Mater. Chem. A.

[43] Zhu F (2019). Off-centered-symmetry-based band structure modulation of hexagonal WO_3_. J. Phys. Condens. Matter..

[44] Moos R, Gnudi A, Härdtl KH (1995). Thermopower of Sr_1-x_La_x_TiO_3_ ceramics. J. Appl. Phys..

[45] Rowe DM, Min G (1995). α-ln σ plot as a thermoelectric material performance indicator. J. Mater. Sci. Lett..

[46] Ohtaki M, Tsubota T, Eguchi K, Arai H (1996). High-temperature thermoelectric properties of (Zn_1−x_Al_x_)O. J. Appl. Phys..

[47] Patel KJ, Panchal CJ, Kheraj VA, Desai MS (2009). Growth, structural, electrical and optical properties of the thermally evaporated tungsten trioxide (WO_3_) thin films. Mater. Chem. Phys..

[48] Cain TA, Kajdos AP, Stemmer S (2013). La-doped SrTiO_3_ films with large cryogenic thermoelectric power factors. Appl. Phys. Lett..

[49] Wang H (2012). Thermoelectric properties of Ti-doped WO_3_ ceramics. J. Mater. Sci. Mater. Electron..

[50] Dong X, Gan Y, Peng S, Dong L, Wang Y (2013). Enhanced thermoelectric properties of WO_3_ by adding SnO_2_. J. Mater. Sci. Mater. Electron..

[51] Yuan H (2009). High-density carrier accumulation in ZnO field-effect transistors gated by electric double layers of ionic liquids. Adv. Funct. Mater..

[52] Sato T, Masuda G, Takagi K (2004). Electrochemical properties of novel ionic liquids for electric double layer capacitor applications. Electrochim. Acta.

