# Supplementary material for: Electrical mapping of thermoelectric power factor in WO3 thin film
Source: Sci Rep. 2022 May 3;12:7202. doi: 10.1038/s41598-022-10908-3 (PMC9065146; doi:10.1038/s41598-022-10908-3)
Supplement: Supplementary file 1 — Supplementary Information. [file 41598_2022_10908_MOESM1_ESM.pdf]

Supplementary Information for "Electrical mapping of thermoelectric power factor in WO_3 thin film"

Sunao Shimizu[1,*], Tomoya Kishi[2], Goki Ogane[2], Kazuyasu Tokiwa[2,*], and Shimpei Ono[1]

[1]Materials Science Division, Central Research Institute of Electric Power Industry (CRIEPI), Kanagawa 240-0196, Japan

[2]Faculty of Advanced Engineering, Tokyo University of Science, Tokyo 125-8585, Japan

[*]s-sunao@criepi.denken.or.jp, tokiwa@rs.tus.ac.jp

Supplementary Notes

Temperature calibration for Seebeck effect measurements

Figure S2 shows the device structure adopted in this study. The temperature gradient is formed along the FET channel by applying electrical heater current I_H to the heater electrode. The two resistive thermometers TH1 and TH2 were prepared to monitor the change of the local temperatures on the close and far sides of the FET channel to the heater, respectively.

We first measured the four-probe resistances R_{Hot} and R_{Cold} for the thermometers TH1 and TH2, respectively. Figure S3 shows the temperature T dependence of R_{Hot} and R_{Cold}, which was measured by changing T of the sample holder in the cryostat. The values of T of the sample holder were monitored using the Cernox thermometers, which was calibrated by Lake Shore Cryotronics, and the model 331 cryogenic temperature controller (Lake Shore Cryotronics). The values of R_{Hot} and R_{Cold} were measured at each T after confirming that the thermal equilibrium was reached. Then, we evaluated the T derivative of R_{Hot} and R_{Cold} as α_{Hot} and α_{Cold}, respectively, as a function of T.

After those calibration of TH1 and TH2, I_H was applied to induce the thermal gradient on the channel. At a fixed T, R_{Hot} and R_{Cold} were measured for several values of I_H, and the increases in R_{Hot} and R_{Cold} due to the local Joule heating were converted into the temperature differences ΔT_{Hot} and ΔT_{Cold} with[1–3]

$$\Delta T_{Hot(Cold)}\,(I_H) = [R_{Hot\,(Cold)}\,(I_H) - R_{Hot\,(Cold)}\,(0)]/\alpha_{Hot\,(Cold)}\,.$$

Therefore, the temperature deference ΔT along the FET channel between TH1 and TH2 was evaluated as $\Delta T\,(I_H) = \Delta T_{Hot}\,(I_H) - \Delta T_{Cold}\,(I_H)$, as shown in Figure 2(a).

The Seebeck coefficient S was estimated as

$$S = -\frac{E}{|\nabla T|} \sim -\left(\frac{\Delta V}{a}\right) / \left(\frac{\Delta T}{b}\right) = -\frac{\Delta V}{\Delta T} \times \frac{b}{a} \ ,$$

where ΔV is the thermoelectric voltage between the source and drain electrodes (see Figure S2), a is the channel length, and b is the distance between TH1 and TH2. The values of a and b are 300 μm and 450 μm, respectively.

Seebeck effect measurement on Bi thin film

To demonstrate the reliability of our experimental procedure mentioned above, we performed the Seebeck effect measurement on a Bi thin film as a reference material.

The Bi thin film with the thickness of 80 nm was synthesized by vacuum evaporation at a pressure of 10^{-5} Torr, which was comparable to the vacuum level in the previous study[4]. We used 99.999% pure Bi, which was purchased from Kojundo Chemical Laboratory. The thin film was prepared on a glass substrate (Corning Eagle XG) having a root-mean-square for surface roughness of less than 1.5 nm. The Bi thin film was patterned by a standard photolithography technique to form the same device structure with that of WO_3 in Figure S2.

Figure S4 shows the ΔV-ΔT plot for the Bi thin film at 295 K. The values of ΔV linearly increased with ΔT, indicating that the thermoelectric effect was correctly measured. The Seebeck coefficient was evaluated to be -52.9 μV/K at 295 K. The value is comparable to that reported for Bi thin films at room temperature in the previous study, assuring the reliability of our measurements[4].

Supplementary Figures

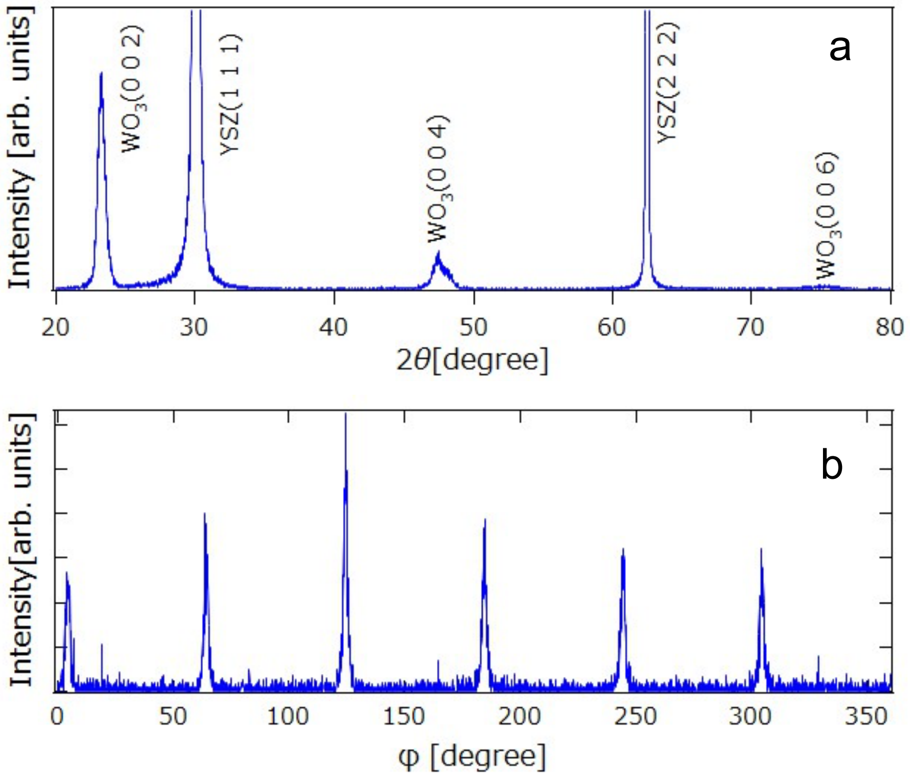

Supplementary Figure S1. XRD spectra of hexagonal WO_3 thin film on YSZ(111) substrate. (a) Out-of-plane XRD (2theta-omega scan) spectra. Only (00*l*) peaks of the WO_3 film were observed, indicating that the WO_3 film was c-axis orientated. (b) Phi scan of (100) WO_3 peak measured by in-plane XRD. The measurement was performed by fixing the angle of the incident X-rays concerning the substrate surface at a shallow $\omega = 0.3°$. The angle of $2\theta\chi$ was fixed at 14.0°, which is the diffraction angle of the (100) plane of WO_3, and the φ axis was rotated from 0° to 360°. The 6-fold hexagonal symmetry of the film (blue trace) was clearly demonstrated. The extracted *a*-axis and *c*-axis parameter were 0.730 and 0.765 nm, respectively.

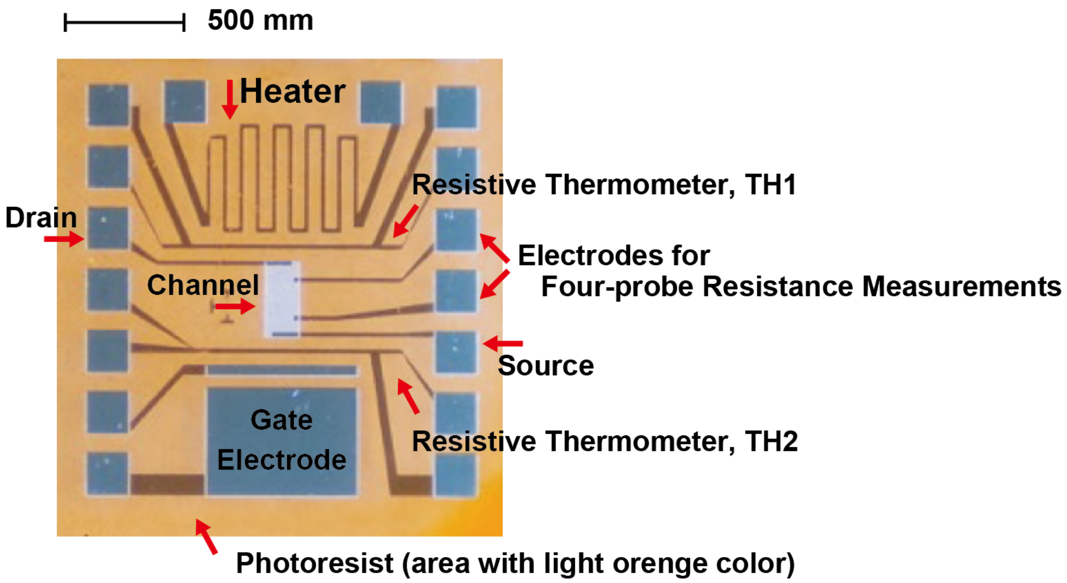

Supplementary Figure S2. Device structure without ionic liquid. The structure in Figure S2 is the same with that in Figure 1b, but Ionic liquid and metal wires are removed to clearly indicate the roles of each electrode. See Experimental Section for the device fabrication process.

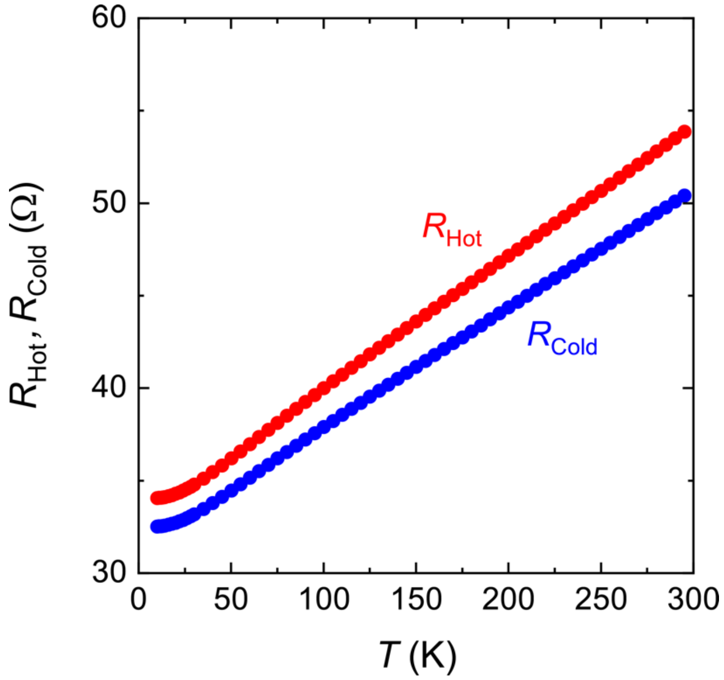

Supplementary Figure S3. Four-prove resistance of resistive thermometers. R_{Hot} and R_{Cold} are the resistances for the thermometers TH1 and TH2, respectively (see Figure S2).

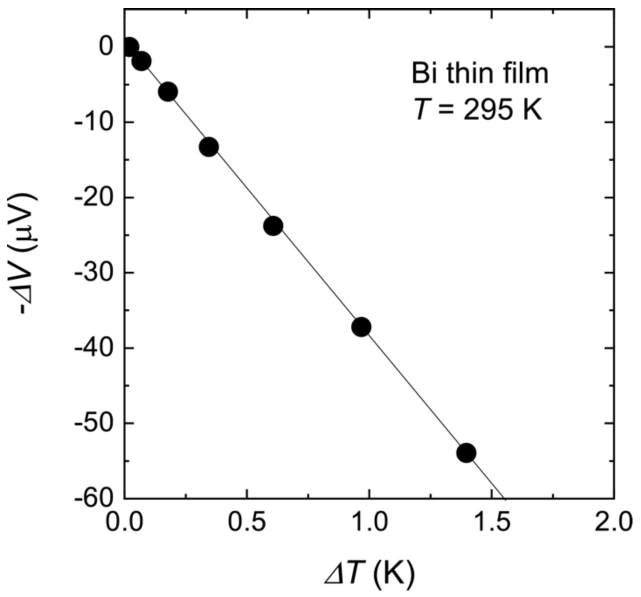

Supplementary Figure S4. Seebeck effect of Bi thin film at 295 K. The thermoelectric power ΔV was measured with increasing the temperature difference ΔT. The values of ΔV was proportional to ΔT.

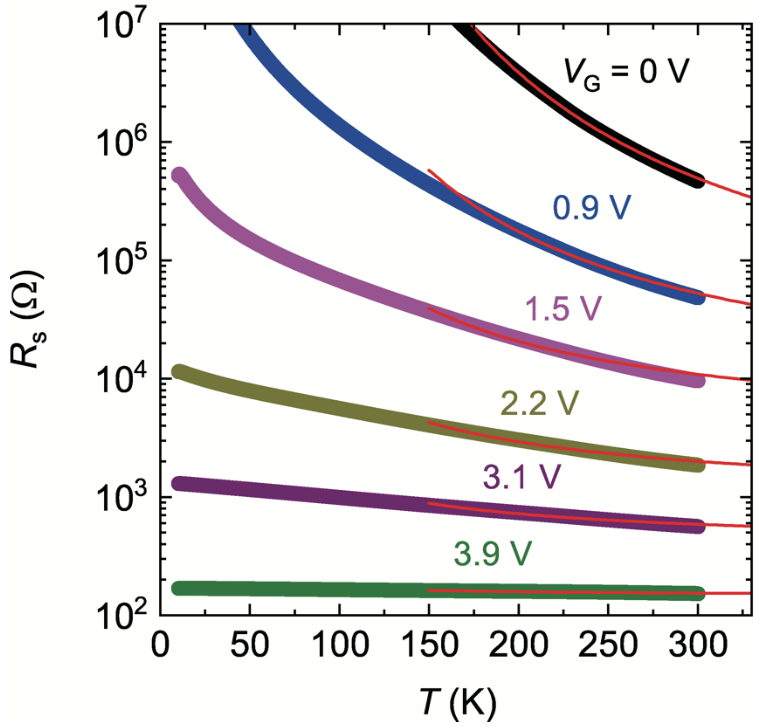

Supplementary Figure S5. Fitting of temperature T dependence of sheet resistance R_s for different gate voltage V_G. Red lines are best fits to the Arrhenius equation, Equation 1. The data above 150 K was used for the fitting to estimate the activation energy E_a, following the analysis by Mattoni *et al*[5]. It was reported that the T dependence of R_s at low temperatures is explained by taking the Mott variable range hopping into account[5]. The value of E_a for V_G = 0 V was estimated to be ~106 meV, which suggests that a shallow donor level exists below the conduction band bottom. With increasing V_G, E_a reduces from ~62 meV for V_G = 0.9 V to ~1 meV for V_G = 3.9 V.

Supplementary Reference

1. Shi, L. *et al.* Measuring thermal and thermoelectric properties of one-dimensional nanostructures using a microfabricated device. *J. Heat Transfer* **125**, 881–888 (2003).
2. Moon, J., Kim, J., Chen, Z. C. Y., Xiang, J. & Chen, R. Gate-Modulated Thermoelectric Power Factor of Hole Gas in Ge-Si Core-Shell Nanowires. *Nano Lett.* **13**, 1196–1202 (2013).
3. Curtin, B. M., Codecido, E. a, Krämer, S. & Bowers, J. E. Field-effect modulation of thermoelectric properties in multigated silicon nanowires. *Nano Lett.* **13**, 5503–8 (2013).
4. Das, V. D. & Soundararajan, N. Size and temperature effects on the Seebeck coefficient of thin bismath films. *Phys. Rev. B* **35**, 5990–5996 (1987).

5. Mattoni, G., Filippetti, A., Manca, N., Zubko, P. & Caviglia, A. D. Charge doping and large lattice expansion in oxygen-deficient heteroepitaxial WO_3. *Phys. Rev. Mater.* **2**, 053402 (2018).
